# Spin to orbital angular momentum transfer in frequency up-conversion

**DOI:** 10.1515/nanoph-2021-0493

**Published:** 2021-11-08

**Authors:** Braian Pinheiro da Silva, Wagner T. Buono, Leonardo J. Pereira, Daniel S. Tasca, Kaled Dechoum, Antonio Z. Khoury

**Affiliations:** Instituto de Física, Universidade Federal Fluminense, 24210-346 Niterói, RJ, Brazil; School of Physics, University of the Witwatersrand, Private Bag 3, Johannesburg 2050, South Africa

**Keywords:** nonlinear optics, OAM, second harmonic generation, structured light

## Abstract

We demonstrate the spin to orbital angular momentum transfer in frequency upconversion with structured light beams. A vector vortex is coupled to a circularly polarized Gaussian beam in noncollinear second harmonic generation under type-II phase match. The second harmonic beam inherits the Hermite–Gaussian components of the vector vortex; however, the relative phase between them is determined by the polarization state of the Gaussian beam. This effect creates an interesting crosstalk between spin and orbital degrees of freedom, allowing the angular momentum transfer between them. Our experimental results match the theoretical predictions for the nonlinear optical response.

## 1 Introduction

The interplay between spin and orbital angular momentum in nonlinear wave mixing has become an active research field. Many recent works have been devoted to the investigation of the crosstalk between different degrees of freedom in nonlinear processes. In our group we have investigated polarization controlled switching of orbital angular momentum (OAM) operations [1, 2], radial-angular coupling in type-II second harmonic generation (SHG) [3, 4], and selection rules in optical parametric oscillation [5, 6]. This subject has revealed a rich and fruitful research field with a much broader scope [7], [8], [9], [10]. For example, it has been used for spiral phase contrast imaging, leading to a visible edge enhancement with invisible illumination [11]. The role played by different kinds of mode structures [12], [13], [14], [15], [16] and controlled phase matching [17] in parametric processes has been widely discussed. Beyond second harmonic generation, the nonlinear response to structured light fields has been also investigated in four-wave mixing [18], [19], [20], plasma [21], surface science [22, 23] and magnetic structures [24]. The generation of angular momentum supercontinuum in a ring array of coupled optical fibers has also been investigated [25]. The quantum optical description of the interplay between polarization and transverse mode structures in parametric down-conversion has been considered long ago [26, 27]. More recently, this description was applied to parametric down-conversion of vector vortex beams both for spontaneous [28] and stimulated [29] processes. The quantum optical approach to the interaction between structured light and nonlinear media has potential applications to novel quantum communication schemes [30].

Much of these developments were made possible by the usage of new optical tools for shaping the phase and polarization distributions of a paraxial beam. Spatial light modulators (SLM) are nowadays a powerful tool for shaping the phase profile and control the diffraction properties of laser beams. They allow for easy and efficient generation of optical vortices, for example. Moreover, the development of specially fabricated plates, capable of shaping the polarization distribution of an optical beam, has made it possible to couple the spin and orbital angular momentum of a light beam. All these developments gave rise to the so called *structured light*, a modern and important topic which has a number of applications in quantum information [31], communication [32, 33], quantum cryptography [33, 34], optical tweezers [35], optical parametric oscillation [5], fiber optics [36], and many others [37], [38], [39], [40].

Among these interesting structured light beams is the *vector vortex* beam, a spin–orbit nonseparable structure that is useful both in classical [41, 42] and quantum regimes. It can be used to study the crosstalk between spin and orbital angular momentum, as already was demonstrated in optical fiber systems [43], [44], [45] and parametric down-conversion [46]. In this work we demonstrate the spin-to-orbital angular momentum transfer in type-II second harmonic generation under noncollinear configuration. The concept of spin–orbit nonseparable structures plays a central role as the spin-to-orbital angular momentum transfer is assisted by a vector vortex beam, which is nonlinearly mixed with a regular Gaussian beam prepared in an arbitrary polarization state. The interest for this effect is two-fold. First, it can be useful for generating OAM beams at high frequencies, such as extreme ultraviolet or soft X-ray, where spatial modulation is not straightforward [47]. Second, it allows for information transfer between different photonic degrees of freedom at different wavelengths, which can be useful for quantum information networks, where one must be able to connect different physical platforms. For example, while telecomm systems employ radiation at 1550 nm, where fiber losses are minimal, the long living energy levels of nitrogen-vacancy (NV) centers, which are good candidates for quantum memories, interact with visible light at 532 nm [48]. Therefore, transfer of a polarization qubit at 1550 nm to an OAM qubit at 532 nm can be useful for quantum information transfer between telecomm photons and NV-center memories [49, 50].

## 2 Spin–orbit coupling in nonlinear wave mixing

This section describes the three-wave mixing process in noncollinear second harmonic generation under type-II phase match. Let us start by describing the incoming electric field of frequency *ω*, which is compounded by two waves with different wave vectors **k**_1_ and **k**_2_. The corresponding electric field amplitudes are given by

(1a) $$E_{k_{1}}^{\left(\omega \right)}=\left[A_{1x}\left(r\right)\hat{x}+A_{1y}\left(r\right)\hat{y}\right]{\text{e}}^{\text{i}k_{1}\cdot r},$$

(1b) $$E_{k_{2}}^{\left(\omega \right)}=\left(\alpha \hat{x}+\beta \hat{y}\right)A_{2}\left(r\right){\text{e}}^{\text{i}k_{2}\cdot r},$$

where $A_{1x}\left(r\right)$ and $A_{1y}\left(r\right)$ are the transverse structures carried by polarizations *x* and *y*, respectively, of the beam **k**_1_, and $A_{2}\left(r\right)$ is the structure of the beam **k**_2_. The unit vectors $\hat{x}$ and $\hat{y}$ are, respectively, the horizontal and vertical polarization states, weighted by the complex numbers *α* and *β*, which obey the normalization relation |*α*|^2^ + |*β*|^2^ = 1.

**Figure 1:**
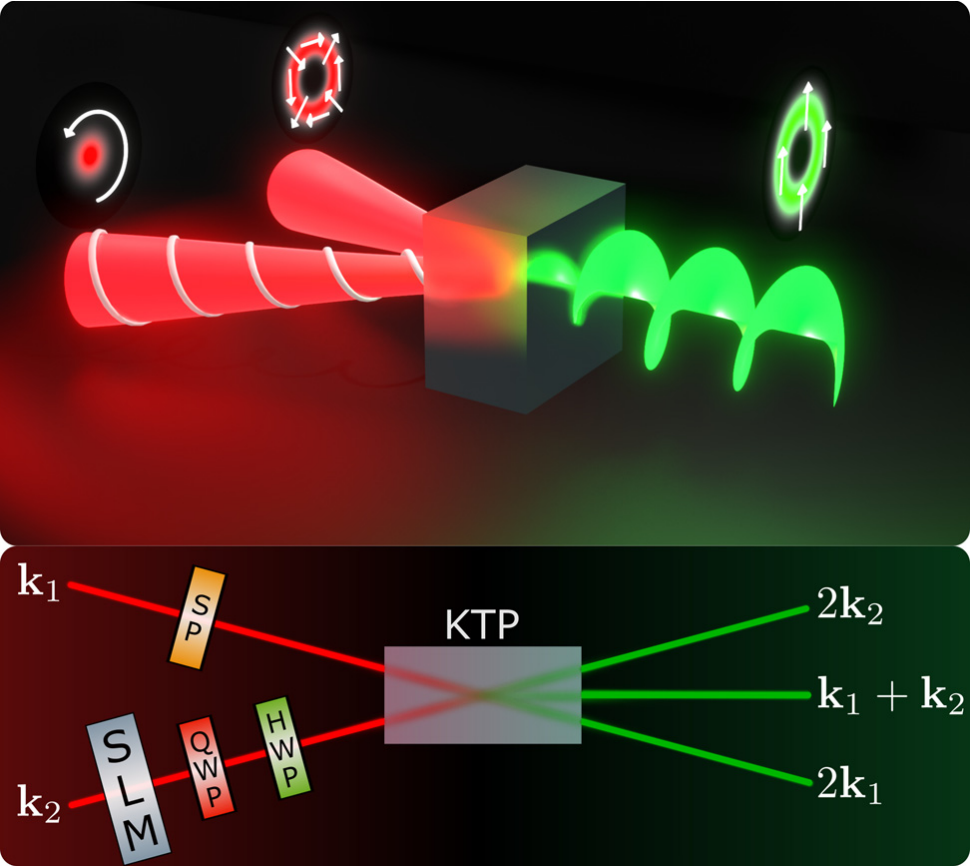
Experimental scheme for spin–orbit angular momentum transfer in second harmonic generation. SP: S-plate for generation of the vector-vortex beam. SLM: spatial light modulator. QWP: Quarter waveplate. HWP: half waveplate. KTP: potassium titanyl phosphate (KTiOPO4) crystal.

Under noncollinear configuration, the output electric field of the second harmonic frequency 2*ω* is formed by three contributions, each one associated with a different combination of the fundamental frequency components. These contributions give rise to three different wave vectors at the second harmonic output: 2**k**_1_, 2**k**_2_, and **k**_1_ + **k**_2_ (shown in Figure 1), which are associated with three simultaneous processes. Wave vectors 2**k**_1_ and 2**k**_2_ are associated with two independent frequency doubling processes of the incoming beams, while **k**_1_ + **k**_2_ corresponds to the nonlinear mixing of the input beams.

In practice, we have three spatially resolved beams that exit the crystal along different directions, which facilitates the independent analysis of their transverse structures. Moreover, if the angle between **k**_1_ and **k**_2_ is small, we can neglect longitudinal components of the input electric fields along the different outgoing directions, as done in reference [2]. The type-II phase match couples the horizontal and vertical polarization components of the fundamental field (*ω*) to generate the second harmonic (2*ω*) with vertical polarization. The resulting electric field amplitudes of the outgoing waves are

(2a) $$E_{2k_{1}}^{\left(2\omega \right)}=g_{1}A_{1x}\left(r\right)A_{1y}\left(r\right){\text{e}}^{\text{i}2k_{1}\cdot r}\hat{y},$$

(2b) $$E_{2k_{2}}^{\left(2\omega \right)}=g_{2}\;\alpha \;\beta \;A_{2}^{2}\left(r\right){\text{e}}^{\text{i}2k_{2}\cdot r}\hat{y},$$

(2c) $$E_{k_{1}+k_{2}}^{\left(2\omega \right)}=\;g_{12}\left[\alpha A_{1y}\left(r\right)+\beta A_{1x}\left(r\right)\right]A_{2}\left(r\right){\text{e}}^{\text{i}\left(k_{1}+k_{2}\right)\cdot r}\hat{y},$$

where *g*_1_, *g*_2_ and *g*_12_ are the coupling coefficients which are proportional to the nonlinear susceptibility of the medium. Here it is important to note that the output wave along **k**_1_ + **k**_2_ carries two contributions. The first one comes from the coupling between the *y* polarization of the input wave **k**_1_ with the *x* polarization of the wave **k**_2_. This term is proportional to the product $\alpha A_{1y}A_{2}$ . The second contribution comes from the coupling between the *x* polarization of the input wave **k**_1_ with the *y* polarization of the wave **k**_2_ and is proportional to the product $\beta A_{1x}A_{2}$ . Therefore, the resulting transverse structure at the output wave is a superposition composed by the transverse modes of input wave **k**_1_, weighted by the polarization coefficients of the input **k**_2_. Provided the transverse structure $A_{2}$ is simply a Gaussian mode, it will only produce a rescaling of the output waist. As we will see, this spin–orbit crosstalk allows the transfer of the spin angular momentum of the input wave **k**_2_ to the orbital angular momentum of the second harmonic output **k**_1_ + **k**_2_.

## 3 Spin-to-orbital angular momentum transfer

The spin–orbit angular momentum transfer becomes evident when we write the input and output modes in terms of Laguerre–Gaussian (LG) and Hermite–Gaussian (HG) functions. The first one is the solution of the paraxial wave equation in cylindrical coordinates. The LG function reads

(3) $$\begin{array}{l} \begin{array}{cc} {LG}_{\ell ,p}\left(\tilde{r},\theta \right) & =R_{\ell ,p}\left(\tilde{r}\right)\;{\tilde{r}}^{\left|\ell \right|}\;{\text{e}}^{\text{i}\ell \theta },\\ R_{\ell ,p}\left(\tilde{r}\right) & =\frac{N_{\ell p}}{w}\;L_{p}^{\left|\ell \right|}\left({\tilde{r}}^{2}\right){\text{e}}^{-\frac{{\tilde{r}}^{2}}{2}}\;{\text{e}}^{-\text{i}\Phi_{N}},\\ \Phi_{N} & =\frac{k\;{\tilde{r}}^{2}}{2R}+\left(N+1\right)\arctan\left(z/z_{0}\right)\;,\\ \;\tilde{r} & =\sqrt{2}\;r/w, \end{array} \end{array}$$

where *N* = 2*p* + |*ℓ*| is the mode order, *ℓ* is the topological charge, *p* the radial order, $L_{p}^{\left|\ell \right|}$ are generalized Laguerre polynomials and $N_{\ell p}$ is a normalization constant. The beam parameters are the wave-front radius *R*, the width *w*, and the Rayleigh length *z*_0_. These modes can carry orbital angular momentum of *ℓℏ* per photon.

The solutions in Cartesian coordinates are the Hermite–Gaussian modes, which are given by

(4) $$\begin{array}{c} {HG}_{m,n}\left(\tilde{x},\tilde{y}\right)=\frac{N_{mn}}{w}\;H_{m}\left(\tilde{x}\right)H_{n}\left(\tilde{y}\right){\text{e}}^{-\frac{{\tilde{x}}^{2}+{\tilde{y}}^{2}}{2}}{\text{e}}^{-\text{i}\Phi_{N}},\\ \tilde{x}=\sqrt{2}\;x/w,\;\tilde{y}=\sqrt{2}\;y/w, \end{array}$$

where $N_{mn}$ is the proper normalization constant, H_*n*_ are the Hermite polynomials with index *n*, and the HG mode order is *N* = *m* + *n*. These modes do not have orbital angular momentum. Both the LG and HG modes constitute orthonormal and complete bases of the transverse mode vector space. In this sense, it is possible to decompose any LG mode in terms of HG modes of the same order, and the opposite is also true [51].

Our reasoning about the spin–orbit angular momentum transfer becomes straightforward when we highlight some multiplicative properties of the HG modes as follows

(5a) $${HG}_{0,0}\left(\tilde{r}\right)\;{HG}_{m,n}\left(\tilde{r}\right)\propto {HG}_{m,n}\left(\sqrt{2}\tilde{r}\right),$$

(5b) $${HG}_{m,0}\left(\tilde{r}\right)\;{HG}_{0,n}\left(\tilde{r}\right)\propto {HG}_{m,n}\left(\sqrt{2}\tilde{r}\right).$$

As we can see, apart from a rescaling of the transverse coordinates, these HG products result in new HG modes that combine the properties of the factor modes. The spin to orbital angular momentum transfer is achieved when specific structures are prepared in the incoming fields with the fundamental frequency *ω*. Let us assume that the input beams with wave vectors **k**_1_ and **k**_2_ are prepared in the following modes

(6a) $$E_{k_{1}}^{\left(\omega \right)}=\left[\sqrt{a}\;{HG}_{0,1}\left(\tilde{r}\right)\;\hat{x}+\sqrt{1-a}{\text{e}}^{\text{i}\phi }\;{HG}_{1,0}\left(\tilde{r}\right)\;\hat{y}\right]{\text{e}}^{\text{i}k_{1}\cdot r},$$

(6b) $$E_{k_{2}}^{\left(\omega \right)}=\left(\sqrt{b}\;\hat{x}+\sqrt{1-b}\;{\text{e}}^{\text{i}\eta }\hat{y}\right)\;{HG}_{0,0}\left(\tilde{r}\right){\text{e}}^{\text{i}k_{2}\cdot r}.$$

Using the HG product properties given by Eqs. (5a) and (5b) for this configuration, the transverse structure of each output beam in the second harmonic frequency will be

(7a) $$B_{1}\left(\tilde{r}\right)=\sqrt{a\left(1-a\right)}{\text{e}}^{\text{i}\phi }{HG}_{1,1}\left(\sqrt{2}\tilde{r}\right),$$

(7b) $$B_{2}\left(\tilde{r}\right)=\sqrt{b\left(1-b\right)}{\text{e}}^{\text{i}\eta }{HG}_{0,0}\left(\sqrt{2}\tilde{r}\right),$$

(7c) $$\begin{array}{ll} B_{12}\left(\tilde{r}\right) & =\sqrt{b\left(1-a\right)}{\text{e}}^{\text{i}\phi }\;{HG}_{1,0}\left(\sqrt{2}\tilde{r}\right)\\ & \;+\sqrt{a\left(1-b\right)}{\text{e}}^{\text{i}\eta }{HG}_{0,1}\left(\sqrt{2}\tilde{r}\right). \end{array}$$

By inspecting these equations, we conclude the following:

1. The transverse structure $B_{1}\left(\tilde{r}\right)$ on output 2**k**_1_ carries a fixed HG_1,1_ mode.
2. The transverse structure $B_{2}\left(\tilde{r}\right)$ on output 2**k**_2_ carries a Gaussian mode HG_0,0_.
3. The transverse structure $B_{12}\left(\tilde{r}\right)$ on output **k**_1_ + **k**_2_ can be any first order mode, controlled by the SU(2) parameters of both input beams.

Therefore, by changing the SU(2) parameters of the incoming beams, it is possible to create any first order transverse mode in the second harmonic. For example, when the **k**_1_ input is a vector beam (*a* = 1/2, *ϕ* = 0) and **k**_2_ is circularly polarized (*b* = 1/2, *η* = ±*π*/2), the spin angular momentum (SAM) *S*_*ω*_*ℏ* per photon on **k**_2_ is transferred to the orbital angular momentum (OAM) *ℓ*_2*ω*_*ℏ* per photon of the output beam **k**_1_ + **k**_2_, that is *ℓ*_2*ω*_ = *S*_*ω*_ = ±1. We next present our experimental results, which confirm this prediction.

## 4 Experimental results

The angular momentum transfer from the spin of the input beam **k**_2_ to the orbital angular momentum of the second harmonic can be shown experimentally with the scheme illustrated in Figure 1. The source of the input beams is a continuous wave (CW) infrared Nd:Yag laser (*λ* = 1064 nm). On **k**_1_, we produce a vector vortex beam using an s-plate (SP – Altechna – Model RPC-1030-02), which comprises a space-variant retarder that converts linear polarization to radial or azimuthal polarization. Its fabrication is based on the inscription of self-organized nanogratings inside fused silica glass using a femtosecond laser. The SU(2) parameters of the vector vortex beam are set to *a* = 1/2 and *ϕ* = 0. The transverse structure and the polarization of **k**_2_ are prepared with a spatial light modulator (SLM) followed by a sequence of a quarter- (QWP) and a half-wave (HWP) plate. The two input beams pass through a potassium titanyl phosphate (KTP) crystal cut for type II phase match, where the nonlinear wave mixing process occurs. All measurements were performed at low pump levels (typically 200 mW), and the SHG quantum efficiency was *P*_2*ω*_/2*P*_*ω*_ ≈ 10^−6^. The results of the second harmonic generation (*λ* = 532 nm) are the three output beams 2**k**_1_, 2**k**_2_, and **k**_1_ + **k**_2_.

**Figure 2:**
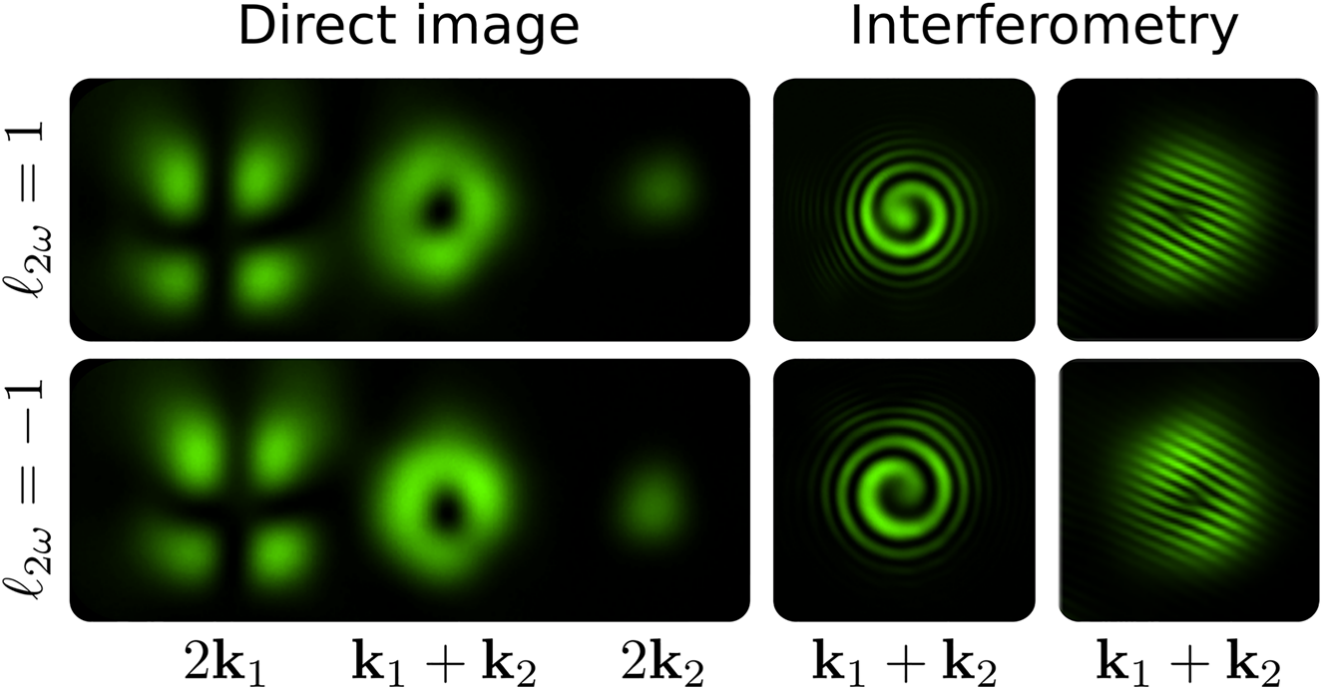
Images produced on the three outputs of the second harmonic field. Laguerre–Gaussian modes with topological charge *ℓ*_2*ω*_ = ±1 are evident in the **k**_1_ + **k**_2_ beam, as characterized by the spiral and forked interference fringes shown on the right.

First, we set the polarization of the input beam **k**_2_ to right circular (*b* = 1/2 and *η* = *π*/2). In this case the output beam **k**_1_ + **k**_2_ acquires a topological charge *ℓ*_2*ω*_ = 1 (LG_1,0_) and carries orbital angular momentum inherited from the spin (*S*_*ω*_ = 1) of the input beam **k**_2_, as illustrated at the top of Figure 1. Then, we switch to left circular polarization (*b* = 1/2 and *η* = −*π*/2), in which case the orbital angular momentum of the **k**_1_ + **k**_2_ output is also switched to *ℓ*_2*ω*_ = *S*_*ω*_ = −1. The corresponding experimental results are shown in Figure 2. The first column is the direct image of the three outputs beams: 2**k**_1_, **k**_1_ + **k**_2_, and 2**k**_2_. The orbital angular momentum of the Laguerre modes is evinced by two different types of interferometry. The first one is the spherical wave interference between the Gaussian mode on 2**k**_2_ and the Laguerre–Gaussian mode on **k**_1_ + **k**_2_. In this case, the OAM is determined by the number and the sense of the spiral fringes. The second measurement employs plane wave interference, in which the orbital angular momentum is given by the fork fringes. The results of Figure 2 confirm the theoretical predictions. The imperfections in the intensity distribution of the modes are mainly due to the walk-off effect present in the wave plates and the nonlinear crystal. Other sources of imperfections are related to limited precision in mode and polarization preparation, and due to small overlaps between the three output beams.

**Figure 3:**
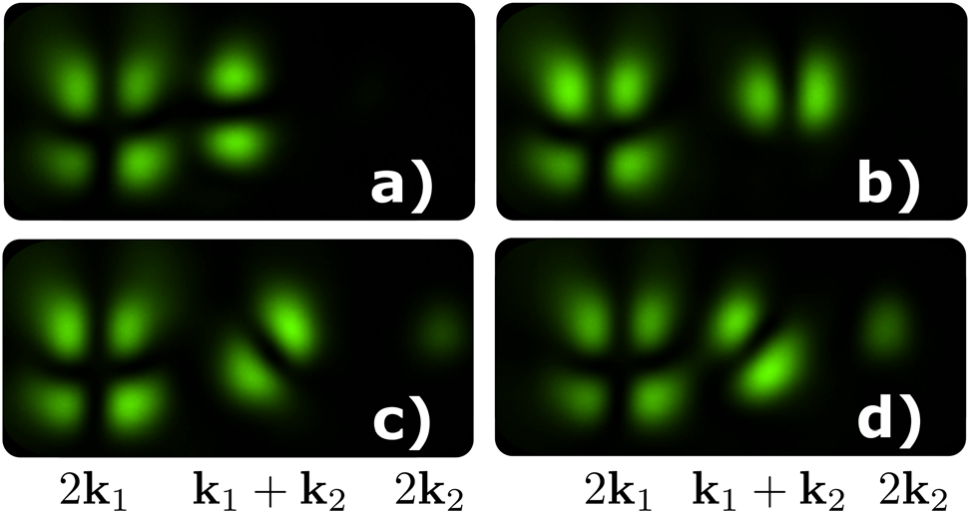
Hermite–Gaussian modes produced on **k**_1_ + **k**_2_ by different orientations of the linear polarization prepared on **k**_2_.

We have also tested the transfer of four different linear polarization states: vertical (*b* = 0 and *η* = 0), horizontal (*b* = 1 and *η* = 0), diagonal (*b* = 1/2 and *η* = 0), and antidiagonal (*b* = 1/2 and *η* = *π*). The experimental results are shown in Figure 3a–d, respectively. The orientations of the Hermite–Gaussian modes produced on **k**_1_ + **k**_2_ are: (a) 88° ± 4°, (b) 1° ± 4°, (c) 51° ± 4°, and (d) −49° ± 4°. They are in good agreement with the theoretical predictions. As expected, when the polarization of the input beam **k**_2_ is either horizontal or vertical, the intensity of the beam 2**k**_2_ vanishes because *b*(1 − *b*) = 0. The small difference between the theoretical and experimental angles, as well as the mode imperfections, has the same origins as before.

If the input beam **k**_2_ carries orbital and spin angular momentum, the output beam **k**_1_ + **k**_2_ will inherit both contributions purely as OAM. In order to demonstrate this, we prepared the input beam **k**_2_ with right circular polarization (*S*_*ω*_ = +1) and transverse mode ${LG}_{\ell_{\omega },p_{\omega }}$ with topological charge *ℓ*_*ω*_. The input beam **k**_1_ was kept in the same vector vortex structure as before. For this configuration the transverse structure of the second harmonic output **k**_1_ + **k**_2_ becomes

(8) $$\begin{array}{ll} B_{12}\left(\tilde{r}\right) & ={LG}_{1,0}\left(\tilde{r}\right)\;{LG}_{\ell_{\omega },p_{\omega }}\left(\tilde{r}\right)\\ & =R_{1,0}\left(\tilde{r}\right)\;R_{\ell_{\omega },p_{\omega }}\left(\tilde{r}\right)\;{\tilde{r}}^{|\ell_{\omega }|+1}\;{\text{e}}^{\text{i}\left(\ell_{\omega }+1\right)\theta }, \end{array}$$

where $R_{\ell ,p}\left(\tilde{r}\right)$ was defined in Eq. (3). The resulting topological charge is *ℓ*_2*ω*_ = *ℓ*_*ω*_ + 1, which corresponds to the added spin and orbital angular momentum of the input beam **k**_1_. Note that if |*ℓ*_*ω*_| + 1 ≠ |*ℓ*_*ω*_ + 1|, then radial rings appear in the second harmonic as a consequence of the radial-angular mismatch discussed in Ref. [4]. In this case, the mode characterization is more involved and falls outside the scope of the present work.

**Figure 4:**
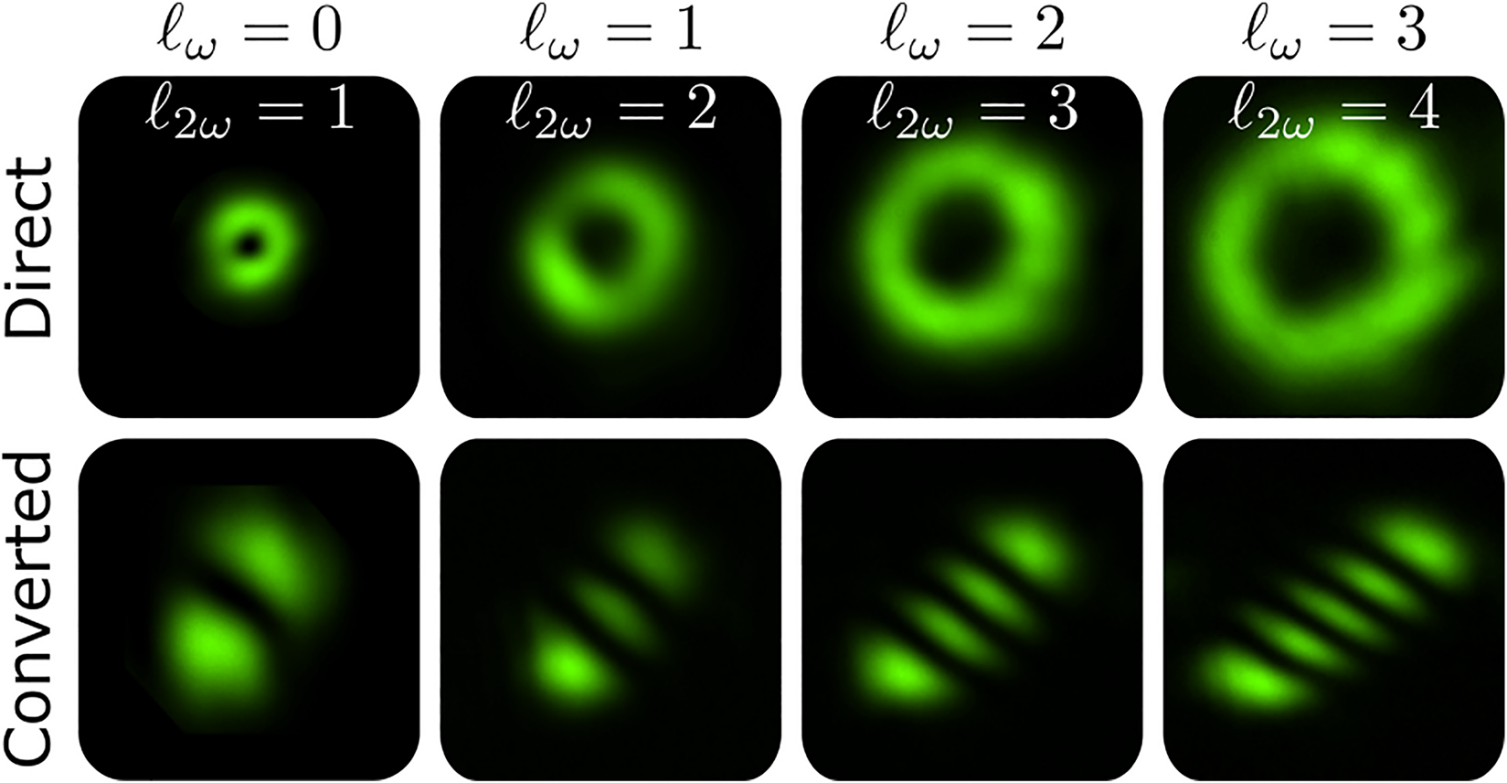
Spin and orbital angular momentum transfer in second harmonic generation with a right circularly polarized **k**_2_ beam (*S*_*ω*_ = +1), carrying topological charges 0 ≤ *ℓ*_*ω*_ ≤ +3 and *p* = 0. Top row displays the direct image acquisition. Bottom row displays the tilted lens conversion for easy identification of the second harmonic topological charge.

**Figure 5:**
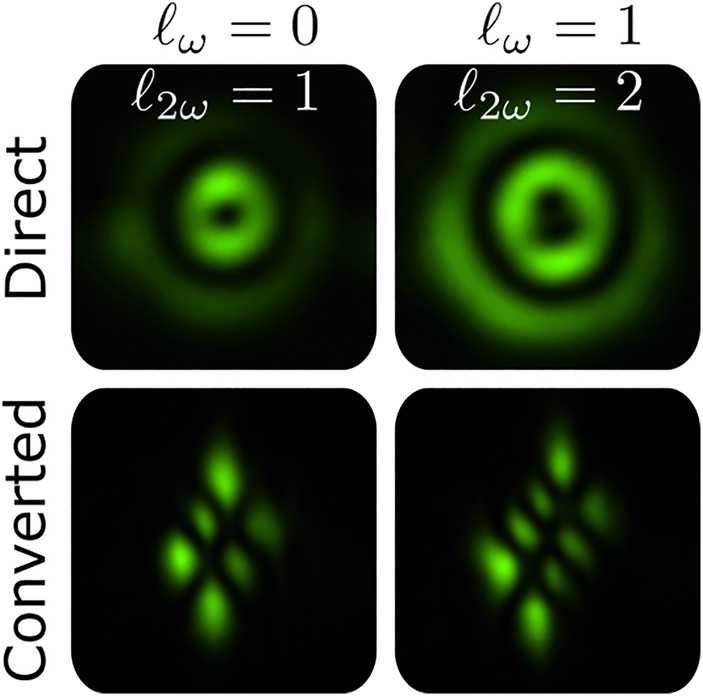
Same as Figure 4 for input beam **k**_2_ prepared in Laguerre–Gaussian modes with 0 ≤ *ℓ*_*ω*_ ≤ 1 and *p* = 1.

We obtained two sets of experimental results that confirm the predictions of Eq. (8). In the first one, the input beam **k**_2_ is prepared in four different Laguerre–Gaussian modes with 0 ≤ *ℓ*_*ω*_ ≤ 3 and *p* = 0. The resulting images are shown in Figure 4. The top images were acquired directly and the bottom ones were registered after a tilted lens mode converter that allows for easy identification of the resulting orbital angular momentum and radial order [52], [53], [54]. The tilted lens analysis is explained in Appendix A. In the second set of experimental results, we prepared two different Laguerre–Gaussian modes with 0 ≤ *ℓ*_*ω*_ ≤ 1 and radial order *p* = 1. The corresponding results are shown in Figure 5. All topological charges measured in both sets of experimental results confirm the theoretical prediction of Eq. (8).

## 5 Conclusions

We demonstrated an interesting effect of spin-to-orbital angular momentum transfer in nonlinear wave mixing of structured light beams. First, we show that an arbitrary polarization state of an input Gaussian beam is transferred to the OAM of the second harmonic frequency when it is mixed with a vector vortex input. The second harmonic beam inherits the Hermite–Gaussian components of the vector vortex, weighted by the polarization coefficients of the Gaussian input. This spin–orbit crosstalk is caused by the type-II phase match condition that couples orthogonal electric field components of the interacting beams. When the Gaussian input beam is replaced by a Laguerre–Gaussian mode carrying a topological charge, both spin and orbital angular momentum are transferred to the second harmonic output, verifying the general relation *ℓ*_2*ω*_ = *S*_*ω*_ + *ℓ*_*ω*_. Our experimental results are in good agreement with the theoretical calculation of the second harmonic field amplitudes. This effect can be useful for generating OAM beams at high frequencies, where spatial modulation is not straightforward [47], and for information transfer between different photonic degrees of freedom at different wavelengths. Moreover, the implementation of quantum information networks requires the ability to connect different physical platforms and to transfer quantum information between physical systems with different energy levels. For example, a polarization qubit state encoded on a telecomm photon (1550 nm) can be transferred to an OAM qubit at 532 nm and finally registered on an NV-center memory [48], [49], [50].

## Figures and Tables

**Figure 6:**
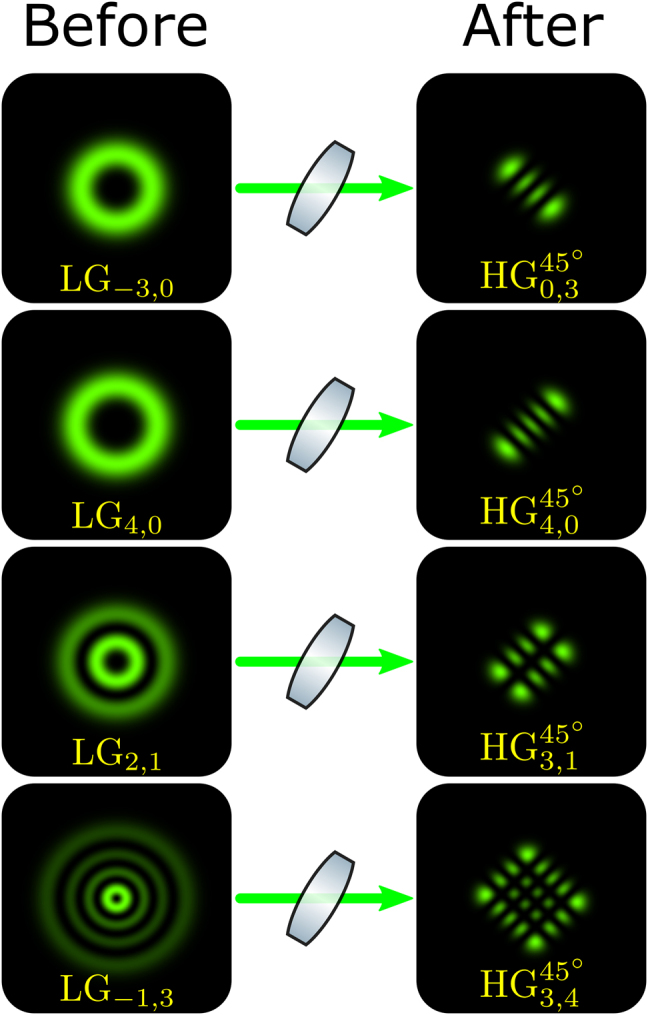
Transverse structure before and after the tilted lens.

## Appendix A: Mode analysis with a tilted lens

The astigmatic transformation implemented by a simple spherical lens with a small tilt angle allows for easy identification of the OAM (*ℓ*) and the radial order (*p*) of pure Laguerre–Gaussian modes [52]. Moreover, it can be used to recognize OAM superpositions [53, 54]. This method is trustworthy and easy to apply to an efficient analysis of orbital angular momentum. An incident Laguerre–Gaussian mode LG_*ℓ*,*p*_ undergoes an astigmatic transformation through the tilted lens and near the focal plane it is converted to a rotated Hermite–Gaussian mode ${HG}_{m,n}^{45{}^{\circ}}$ , where

(9)
$${HG}_{m,n}^{45{}^{\circ}}\equiv {HG}_{m,n}\left(\frac{x+y}{\sqrt{2}},\frac{x-y}{\sqrt{2}}\right).$$

This transformation is illustrated in Figure 6. The orbital angular momentum and radial index of the incident mode are readily determined from

1. *ℓ* = *m* − *n*.
2. *p* = min(*m*, *n*).
